# Irrelevant sights and sounds require spatial suppression: ERP evidence

**DOI:** 10.1111/psyp.14181

**Published:** 2022-09-17

**Authors:** Jessica Lunn, Nick Berggren, Jamie Ward, Sophie Forster

**Affiliations:** ^1^ School of Psychology and Sussex Neuroscience University of Sussex Brighton UK; ^2^ Department of Psychological Sciences Birkbeck, University of London London UK

**Keywords:** attentional capture, distraction, ERPs, pd, suppression, visual search

## Abstract

Both real‐world experience and behavioral laboratory research suggest that entirely irrelevant stimuli (distractors) can interfere with a primary task. However, it is as yet unknown whether such interference reflects competition for spatial attention – indeed, prominent theories of attention predict that this should not be the case. Whilst electrophysiological indices of spatial capture and spatial suppression have been well‐investigated, experiments have primarily utilized distractors which share a degree of task‐relevance with targets, and are limited to the visual domain. The present research measured behavioral and ERP responses to test the ability of salient yet entirely task‐irrelevant visual and auditory distractors to compete for spatial attention during a visual task, while also testing for potentially enhanced competition from multisensory distractors. Participants completed a central letter search task, while ignoring lateralized visual (e.g., image of a dog), auditory (e.g., barking), or multisensory (e.g., image + barking) distractors. Results showed that visual and multisensory distractors elicited a P_D_ component indicative of active lateralized suppression. We also establish for the first time an auditory analog of the P_D_ component, the P_AD_, elicited by auditory and multisensory distractors. Interestingly, there was no evidence to suggest enhanced ability of multisensory distractors to compete for attentional selection, despite previous proposals of a “special” saliency status for such items. Our findings hence suggest that irrelevant multisensory and unisensory distractors are similarly capable of eliciting a spatial “attend‐to‐me” signal – a precursor of spatial attentional capture – but at least in the present data set did not elicit full spatial attentional capture.

## Abstract 2

Our research speaks to two theoretical debates; whether entirely task‐irrelevant stimuli can compete for spatial attention, and the role of endogenous attention in the proposed special saliency status of multisensory stimuli. We demonstrate that irrelevant visual and multisensory distractors elicit an ERP component indicative of active spatial suppression (P_D_) and establish an auditory analog of this component (P_AD_). We also find no evidence of an enhanced ability of multisensory distractors to compete for spatial attention.

## 1 INTRODUCTION

In our daily lives we often find that we are distracted by irrelevant sights and sounds, and this irrelevant distraction can lead to a variety of negative consequences. For example, highly distractible individuals are more at risk of serious accidents (Larson et al., 1997), and distraction by task‐irrelevant stimuli has been found to account for over 10% of vehicle crashes that resulted in driver hospitalization (McEvoy et al., 2007). Despite its apparent ubiquity in daily life, the phenomenon of distraction by entirely irrelevant events was for some time under‐represented by laboratory studies of attention (see Forster, 2013). Motivated by a desire to parallel real‐life distraction, Forster and Lavie (2008a) developed the irrelevant distractor task. This task measures the degree of reaction time slowing to a central letter search task, associated with the presentation of highly salient distractors (cartoon images). These distractors were not only irrelevant to the main search task participants were required to undertake, but also appeared at task irrelevant, off‐centre, locations. The finding that the irrelevant distractors slow down reaction times to a central task has been replicated many times (e.g., Cunningham & Egeth, 2018; Forster et al., 2014; Forster & Lavie, 2008a, 2011, 2014; He & Chen, 2010; Lunn et al., 2019) including in a large sample (Forster & Lavie, 2014), and has also been shown to correlate with clinical symptoms of distraction in adults with Attention Deficit Hyperactivity Disorder (ADHD) and those with trait level symptomology (Forster & Lavie, 2016). As of yet, the irrelevant distractor task has mainly been applied to the visual domain. However, a recent paper has reported a distraction effect employing audiovisual distractors (Lunn et al., 2019), with equivalent results.

While it is now well‐established that entirely irrelevant distractors can slow us down and interfere with our current tasks, it is not known precisely what mechanism underlies this interference. One possibility is that the reaction time slowing reflects the additional time taken for covert orienting to the distractor location and then re‐orienting back to the target (i.e., spatial attentional capture). However, it has long been debated whether spatial attentional capture by entirely irrelevant stimuli is possible, with non‐spatial mechanisms also proposed, for example “filtering costs” (e.g., Folk & Remington, 1998), whereby the distractor does not itself attract spatial attention but delays the deployment of spatial attention toward the target.

Goal‐driven theories of attention argue that attention can be captured by stimuli presented in irrelevant spatial locations, but only if they have a degree of task relevance, such as those that match the attentional set of a search task (e.g., Bacon & Egeth, 1994; Folk & Remington, 1998). On the other hand, some prominent stimulus‐driven theories of attention propose that salient stimuli are able to automatically capture attention regardless of our current goals (e.g., Theeuwes, 1992, 2010), but only if they are inside of our “attentional window” (Belopolsky & Theeuwes, 2010; Theeuwes, 2004), an area of visual space in which task‐relevant stimuli are expected to be found. Taken together, these theories imply that entirely irrelevant distractors, appearing outside of the attentional window, are unable to catch our attention. A recent intermediate account is the “signal suppression hypothesis” (see Luck et al., 2021, for a discussion of all three theories), which proposes that salient stimuli elicit a spatial “attend‐to‐me” signal (e.g., Theeuwes, 1992, 2010), but that this can be actively suppressed to prevent attention being fully allocated to, or captured by, the salient item.

A challenge for demonstrating spatial capture by entirely irrelevant stimuli is that behavioral measures of spatial attention, such as spatial cuing, typically involve stimuli occurring in, or perceptually grouped around, a task‐relevant location (e.g., Folk et al., 1992). Hence, in order to measure spatial capture by irrelevant stimuli without introducing a degree of task relevance, electrophysiological indices of these processes are particularly useful as indirect measures. There are two lateralized Event Related Potential (ERP) components primarily used in this area of research. First, the N2pc is held to reflect attentional selection of items in visual space (Kiss et al., 2008; Luck & Hillyard, 1994). This component is reflected at posterior electrodes, characterized by a negative deflection in the ERP waveform contralateral to a stimulus presented at an attended location, and elicited 200–350 ms post stimulus onset. An analogous component has also been documented for selection of information within the auditory domain, occurring at anterior electrodes ‐ the N2ac (Gamble & Luck, 2011). Salient auditory stimuli have also been demonstrated to activate the visual cortex, eliciting auditory‐evoked contralateral positivity (ACOP), observed over parietal electrodes at approximately 260–360 ms (McDonald, Stormer, et al., 2013). Second, the P_D_ component is the opposite of the N2pc ‐ a contralateral *positivity*, occurring at the same electrode sites from as early as 100 ms (e.g., Gaspelin & Luck, 2018) to as late as 300–400 ms (e.g., Sawaki et al., 2012), which is believed to index spatially localized suppression of a distractor stimulus in response to a spatial “attend‐to‐me” signal (Hickey et al., 2008; Sawaki & Luck, 2010). The size of the P_D_ has been found to correlate with behavioral distractor interference (Gaspelin & Luck, 2018), and also predicts whether or not a distractor elicits an N2pc. McDonald, Green, et al. (2013) found that in visual search trials where responses to a target were slow, a significant N2pc to the distractor was found followed by a marginally significant P_D_ (indicating the two may coexist), but on fast response trials no distractor N2pc was found, with only the P_D_ being elicited instead. Thus, a visual distractor can elicit an “attend‐to‐me” signal and be actively suppressed by a participant, despite not producing a behavioral cost. However, no auditory analog of the P_D_ has, as of yet, been established – and as such it remains unclear whether auditory stimuli can similarly elicit “attend‐to‐me” signals.

Research using both the N2pc and the P_D_ component has typically utilized stimuli which share a degree of task relevance, for example appearing in a potential target location as in the additional singleton paradigm or spatial cuing tasks (e.g., Burra & Kerzel, 2014; Gaspar & McDonald, 2014; Gaspelin et al., 2015; Sawaki & Luck, 2010), or sharing similar features with the target (e.g Burra & Kerzel, 2014; Gaspar & McDonald, 2014; Hickey et al., 2006; Sawaki & Luck, 2010, 2013; Weaver et al., 2017). Indeed, Sawaki and Luck (2010), found that color letter singletons presented in a task‐irrelevant location elicited a P_D_ component when the target stimuli were also letters, but not when the central task was changed to one which did not involve letters. Although the lack of P_D_ in the latter condition was attributed to increased perceptual load of the new task, we note that behavioral research has demonstrated that sharing target features (even if these features are not task‐relevant) confers sufficient relevance to allow interference from distractors in task‐irrelevant locations (Lichtenstein‐Vidne et al., 2012). Whether entirely irrelevant stimuli would still elicit an “attend‐to‐me” signal to capture our attention, which must be suppressed, is hence less well‐established. One recent study adapting Forster and Lavie (2016)'s irrelevant distractor paradigm speaks to the issue of complete irrelevance: Neumann et al. (2018) found that irrelevant distractor images (faces and cars) elicited a P_D_ component. Interestingly, no N2pc to the distractors was found, despite behavioral distractor interference. Hence, this initial evidence suggests that irrelevant visual stimuli may nevertheless elicit a spatial “attend‐to‐me” signal, but not full spatial attentional capture. However, this prior experiment used the original version of the irrelevant distractor task, which involved a perceptually imbalanced display (with the irrelevant distractor on one side and no stimulus on the opposite side). As such, it cannot be fully ruled out that the apparent P_D_ might be alternatively explained by this perceptual asymmetry. In addition, it remains unknown whether these findings would extend to stimuli presented in other, or multiple, modalities.

As noted above, the dominant focus of research into both irrelevant distraction and attentional capture has been in the visual domain, but real‐world sources of distraction often involve other senses, or even a combination of more than one sense. Indeed, multisensory stimuli have been proposed to be especially effective in capturing attention (e.g., Santangelo & Spence, 2007), and it has even been suggested that signals from two different sensory modalities may integrate pre‐attentively (e.g., Bertelson et al., 2000; though see Pápai & Soto‐Faraco, 2017). If it is the case that a multisensory stimulus is a stronger competitor for attention, then we may expect more efficient (faster and/or stronger) spatial attentional capture. Note that in a previous study we have found no evidence of increased distraction by multisensory stimuli when using behavioral measures alone (Lunn et al., 2019). This result initially appeared in line with previous research showing that multisensory integration is compromised when the stimuli involved are unattended (e.g., Alsius et al., 2005; Pápai & Soto‐Faraco, 2017; Talsma et al., 2007). However, behavioral distractor interference may not be a sensitive measure to detect fast‐acting attentional capture by multisensory distractors. Indeed, if both unisensory and multisensory distractors capture attention, but the time course of this capture (and subsequent release) is faster for multisensory stimuli, then this might actually result in less interference for multisensory versus unisensory stimuli. Faster capture accompanied by delayed disengagement could also cancel each other out, resulting in no effect of multisensory presentation on behavioral distraction. Such effects, however, could be captured with time‐resolved neuroimaging measures such as ERPs. Hence, we adapted the present experiment from a multisensory distractor task used in our previous study (Lunn et al., 2019; low perceptual load condition). This approach allowed us to test for capture of attention by irrelevant visual, auditory and multisensory stimuli, and index whether or not faster spatial capture by, or larger spatial suppression of, multisensory distractors is present.

In summary, the primary goal of the present research was to provide a direct test of spatial capture by entirely irrelevant visual, auditory, and multisensory stimuli. A contralateral negativity to these distractors (N2pc/N2ac) would signify the selection of these distractors in the visual or auditory space, and a contralateral positivity (P_D_) would indicate that they do elicit a spatially localized “attend‐to‐me” signal indicative of necessitating active suppression. A contralateral positivity to auditory distractors would also be the first evidence of an auditory analog to the P_D_. Our second goal was to compare effects across visual, auditory and multisensory distractors. In particular, if multisensory stimuli are more effective at capturing attention, then we may expect a faster time course, or increased amplitude, of these components for multisensory compared to either unisensory distractor stimuli.

## 2 METHOD

### 2.1 Participants

Eighteen participants (15 female) aged between 18 and 24 years (M = 21, SD = 2) were recruited at the University of Sussex to participate in this experiment. Participants either gained course credits, or were paid, to take part. Data from three participants were excluded and replaced, two due to excessive movement artifacts, and one due to technical issues with the electrophysiological recording. The study was approved by the Sciences and Technology Research Ethics Committee (C‐REC) at the University of Sussex. All participants reported normal or corrected‐to‐normal vision and hearing, and no known skin, neck or head problems. Sample size calculations were conducted prior to data collection, using G*Power software (Faul et al., 2009), revealing that to detect an effect size of ${\eta_{p}}^{2}$ = .37 (α = .05, 1−β = .99), a sample size of 16 was required. This effect size was chosen from the main effect of electrode laterality (contralateral vs ipsilateral) in Hickey et al.'s (2008) Experiment 1 for the P_D_, and would also sufficiently power the behavioral distraction effect (*d* = 4.17 based on Forster and Lavie's (2016) low load condition), and measurement of the N2pc (${\eta_{p}}^{2}$ = .55 based on the main effect of laterality in Hickey et al.'s, 2006 Experiment 1) and N2ac (${\eta_{p}}^{2}$ = .50 based on the overall N2ac in Gamble and Luck (2011)).

### 2.2 Stimuli and procedure

The experiment was programmed and presented using E‐Prime v2.0, on a 19‐inch CRT monitor (resolution 1600 × 1200) at a refresh rate of 85 Hz and at a viewing distance of approximately 57 cm. Auditory stimuli were delivered over Trust Leto 2.0 speakers positioned on the left and right side of the screen.

**FIGURE 1:**
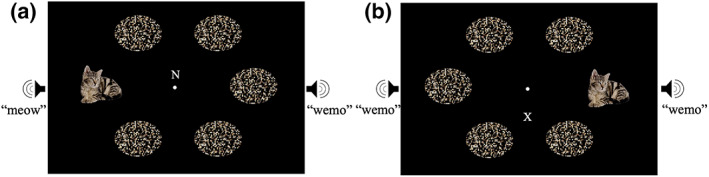
Example stimulus displays, not to scale. X/N letter identification along the vertical midline. (a) Multisensory cat distractor with scrambled cat images and scrambled cat sound from the speaker on the opposite side, (b) visual cat distractor with scrambled cat images and scrambled cat sound from both speakers.

The task was adapted from the multisensory irrelevant distractor paradigm as used in Lunn et al. (2019) (Figure 1). As in this task, trials began with a central fixation point (white, radius 0.1°) presented over black background for 100 ms, followed by a 500 ms stimulus display. Participants were required to identify a target letter (X or N; white; subtending 0.4° × 0.5°) presented centrally on screen, responding with one of two key presses to indicate which letter they had seen. Participants were instructed to ignore anything that was presented elsewhere on the screen. Distractor stimuli were presented with equal likelihood in the left and right side of the visual field, or from the left or right speaker, with each of three distractor types (visual, auditory, multisensory) being presented on 8% of trials. Visual distractors were identical to those previously used in our earlier study, consisting of a photograph of an animal, randomly selected from six possible images (dog, cat, pig, horse, cow, sheep). In order to produce robust distractor effects in this paradigm it is important to use stimuli that are both salient and meaningful **(**Forster & Lavie, 2008b), other versions of this task, have employed meaningful images such as cartoons, faces, and food (e.g., Cunningham & Egeth, 2018; Forster et al., 2014; Forster & Lavie, 2008a, 2008b, 2011, 2016; He & Chen, 2010). Animal stimuli were chosen in our previous behavioral version of this paradigm, because the semantic congruence between vision and audition should facilitate multisensory integration (see e.g., Laurienti et al., 2004). Images were presented in full color with a black background, subtending 5.0° to 5.6° vertically, by 4.5° to 6.3° horizontally. Auditory distractors consisted of characteristic animal sounds (same animals as the images) presented from one of the speakers (600–1000 ms in duration). Multisensory distractors consisted of the simultaneous presentation of the animal image presented with its corresponding sound, both presented from the same side.

Changes were made to the behavioral task established in Lunn et al. (2019), to allow for measurement of the N2pc/N2ac and P_D_. In the present experiment the target letters were presented at one of 6 possible locations along the vertical meridian of the screen (either 1.0°, 2.5°, or 4.0° above and below the central fixation point) rather than arranged in the shape of a circle, so that targets were not presented laterally. Furthermore, on each trial, 6 images arranged in an imaginary circle (radius 9°) were displayed in the periphery. In the no distractor trials all 6 images were identical scrambled images of an animal (scrambled in 4 × 4 pixel blocks; selected randomly from dog, cat, cow, horse, pig, or sheep) subtending 4.4° vertically, by 6.2° horizontally. At the same time, the scrambled noise of this animal (produced in MATLAB R2018b by shuffling a matrix of the audio data while maintaining the same sampling rate) was presented from both the left and right speaker. Visual distractor images, when present, were displayed at the 3 o'clock or 9 o'clock position instead of the scrambled image of the same animal. Auditory distractor sounds consisted of the characteristic noise of the animal instead of the scrambled sound from one of the loudspeakers, whilst the scrambled noise of the same animal played from the other speaker (approximately 67 dB). Multisensory distractors consisted of a combination image and sound, which was both spatially and semantically congruent. Scrambled and non‐scrambled images of the same animal were approximately equiluminant (average 21.04 based on the CIELAB color space, measured using the SHINE_color toolbox for MATLAB; Dal Ben, 2019). The scrambled images and sounds were added to reduce low‐level perceptual asymmetry between the two sides of the display. As it was particularly important to avoid eye movements in the present task, the central fixation point remained on screen throughout the entire block, to avoid an additional offset/onset, and the inter‐trial interval was jittered (1300–1800 ms) in order to make habituation to the displays less likely.

Participants completed three slowed down, example trials (i.e., stimuli remained on screen until response), followed by 12 practice trials. They then completed 12 blocks of 100 trials for a total of 1200 trials, of which 96 contained auditory distractors, 96 visual distractors and 96 multisensory distractors. Distractors were presented randomly throughout blocks, excluding the first three trials which were warm‐up trials and always had no distractor.

### 2.3 ERP recording and analysis

Electrical brain activity was continuously digitized from an array of 64 Ag/AgCl electrodes mounted in an elastic cap arranged according to the 10–20 system, at a 1000 Hz sampling rate using an ANT Neuro amplifier. Horizontal EOG was also recorded bipolarly using electrodes placed at the outer canthi of both eyes, and vertical EOG from the inferior and superior orbit of the left eye. Impedances were monitored throughout the recording session and kept consistently low, typically <10kΩ. Data processing was conducted using EEGlab (Delorme & Makeig, 2004) and ERPlab (Lopez‐Calderon & Luck, 2014). Bad channels were interpolated using EEGlab's spherical spline interpolation function (<1% of total channels, none of which were those used in the main analyses). Following referencing to the average of the left and right mastoids, the following filters were applied: 0.1 Hz (12 db/oct; zero phase) high‐pass, 30 Hz (24 db/oct; zero phase) low‐pass, 50 Hz notch filter (to remove line noise) and an 85 Hz notch filter (to remove line noise specifically caused by the speakers, visual inspection of the data confirmed that the notch filter did not create or remove components). Epochs were baseline corrected according to a 100 ms pre‐stimulus presentation window and neural activity was examined for 500 ms post‐stimulus presentation. Automatic offline artifact rejection was performed by removing epochs contaminated by eyeblinks (peak‐to‐peak threshold ±75 μV) and eye movements (step‐like artifacts threshold ±30 μV), at VEOG and HEOG, respectively. Where there were technical issues with the recording from these electrodes, Fp1 was used and epochs were manually inspected (*n* = 2 in each case). Given the importance of removing all HEOG activity when measuring the N2pc or the P_D_, we note that all statistical analyses remain significant with the exclusion of the two participants for whom the recording was affected. In all remaining participants, we assessed whether residual HEOG activity remained after artifact rejection during our critical time window (100–300 ms), by comparing mean HEOG amplitude on trials where the distractor was presented in the left versus right hemifield, separately for all three distractor types. No systematic differences were present (*t*s <1.38, *p*s > .189). Epochs contaminated by drifts, blocked electrodes or muscle‐related potentials (±200 μV at all other electrodes) were also rejected. No correct distractor trials had RTs of less than 100 ms, so none were excluded on this basis. All trials in which participants made incorrect responses were also excluded, leaving on average 68.90% (SD = 15.99, range = 40–88%) of distractor trials to be analyzed. PO7/PO8 electrodes where the N2pc and P_D_ are typically maximal were chosen a priori to be examined for these components for both visual and multisensory distractor conditions. N2ac was found at a cluster of anterior electrode sites (Gamble & Luck, 2011) which we examined for the presence of this component, or an auditory analog of the P_D_, for both auditory and multisensory distractor conditions.

The N2pc and P_D_ components have been found to vary substantially in their onset latency. The N2pc typically occurs approximately 200–350 ms after stimulus onset (Kiss et al., 2008), and the P_D_ was first found occurring at approximately 220–280 ms (Hickey et al., 2008), but has since been shown as early as 100–220 ms (Gaspelin & Luck, 2018), therefore we chose a wider time window of 100–300 ms to test for the presence of this component. Following previous research, we confirmed the amplitude analyses using a non‐parametric permutation method developed by Sawaki et al. (2012). In this approach, the distribution of area amplitude that could be expected from noise alone is estimated from random permutations of the data, and no assumption is required with regard to normality or equal variance (Gaspelin & Luck, 2018; Sawaki et al., 2012, 2017; Sawaki & Luck, 2013). To perform the permutation test, the side of the distractor was randomly recoded for every trial and participant, and ERPs were re‐averaged to a grand average. Positive area value was then measured between 100–300 ms, at PO7/PO8 for the visual and multisensory distractors, and at the averaged anterior electrode cluster for the auditory and multisensory distractors. This was repeated 500 times, resulting in a null distribution. If the observed area amplitude from the original data is greater than 95% of the values in the null distribution, then the P_D_ can be considered significant. To compare the P_D_ and P_AD_ elicited by multisensory versus unisensory distractors, the same method was used, but it was the modality of the distractor that was randomly recoded, with the laterality kept the same.

The first 3 trials from each block, and any in which RT was less than 100 ms, were excluded from behavioral analysis of the central task. The Greenhouse–Geisser correction for non‐sphericity was used for ANOVAs with factors containing more than two levels. The uncorrected degrees of freedom, but corrected *p* values, are reported.

## 3 RESULTS

Data can be downloaded from the Open Science Framework (osf.io/yv6rg/).

### 3.1 Behavioral results

A one‐way within‐subjects ANOVA with the factor of distractor type (multisensory, unisensory auditory, unisensory visual, no distractor) on RT in correct trials, revealed no main effect of distractor (*F*[3, 51] = 1.88, *p* = .157, ε = .81, η^2^ = .10). Unexpectedly, and in contrast to our earlier findings, no evidence of behavioral distraction was found: RTs were no slower in the presence of any of the three distractor types compared with no distractor trials (*t* < 1, B_H(0,23)_ = .34 for multisensory distractors; *t* < 1, B_H(0,23)_ = .09 for auditory distractors; *t*(17) = 1.57, *p* = .067, B_H(0,17)_ = 1.68 for visual distractors), nor were multisensory distractors any more distracting than either of the two unisensory types *(t*(17) = 1.29 *p* = .108, B_H(0,23)_ = .84 and *t* < 1, B_H(0,23)_ = .12 for multisensory compared with auditory and visual, respectively). We note that the present experiment is substantially longer than previous studies employing this paradigm, which typically employ 4 blocks for each load condition (e.g., Forster & Lavie, 2008a, 2008b; Lunn et al., 2019). To rule out the possibility that the distractor effect was present at the start of the experiment but reduced across its duration, we also ran these analyses on the first four blocks only. Again, no main effect of distractor was found (*F*[3, 51] = 1.22, *p* = .310, ε = .74, η^2^ = .07), nor a statistically significant distractor effect for any of the three distractor types (*p*s > .051, see Supplementary Materials for the descriptive data).

A one‐way within‐subjects ANOVA with the factor distractor type (multisensory, unisensory auditory, unisensory visual, no distractor) on percentage error rate, revealed a main effect of distractor type (*F*[3, 51] = 3.62, *p* = .035, η^2^ = .18, ε = .70) (Table 1). Two‐tailed follow up *t* tests revealed that this was reflected by a small yet statistically reliable effect, where error rates were in fact lower in the presence of a multisensory or auditory distractor compared to when no distractor was present (*ps* < .002), but not when a visual distractor was present (*p* = .176).

**TABLE 1:** Mean RTs (SE in parentheses) and error rates (%) as a function of load and distractor type

	Distractor condition			
	Multisensory	Auditory	Visual	No distractor
RT(ms)	551 (26)	545 (27)	556 (28)	548 (25)
% Error	7.58	6.67	8.06	9.22

### 3.2 Electrophysiological results from distractor trials

#### 3.2.1 Visual and multisensory distractors

**FIGURE 2:**
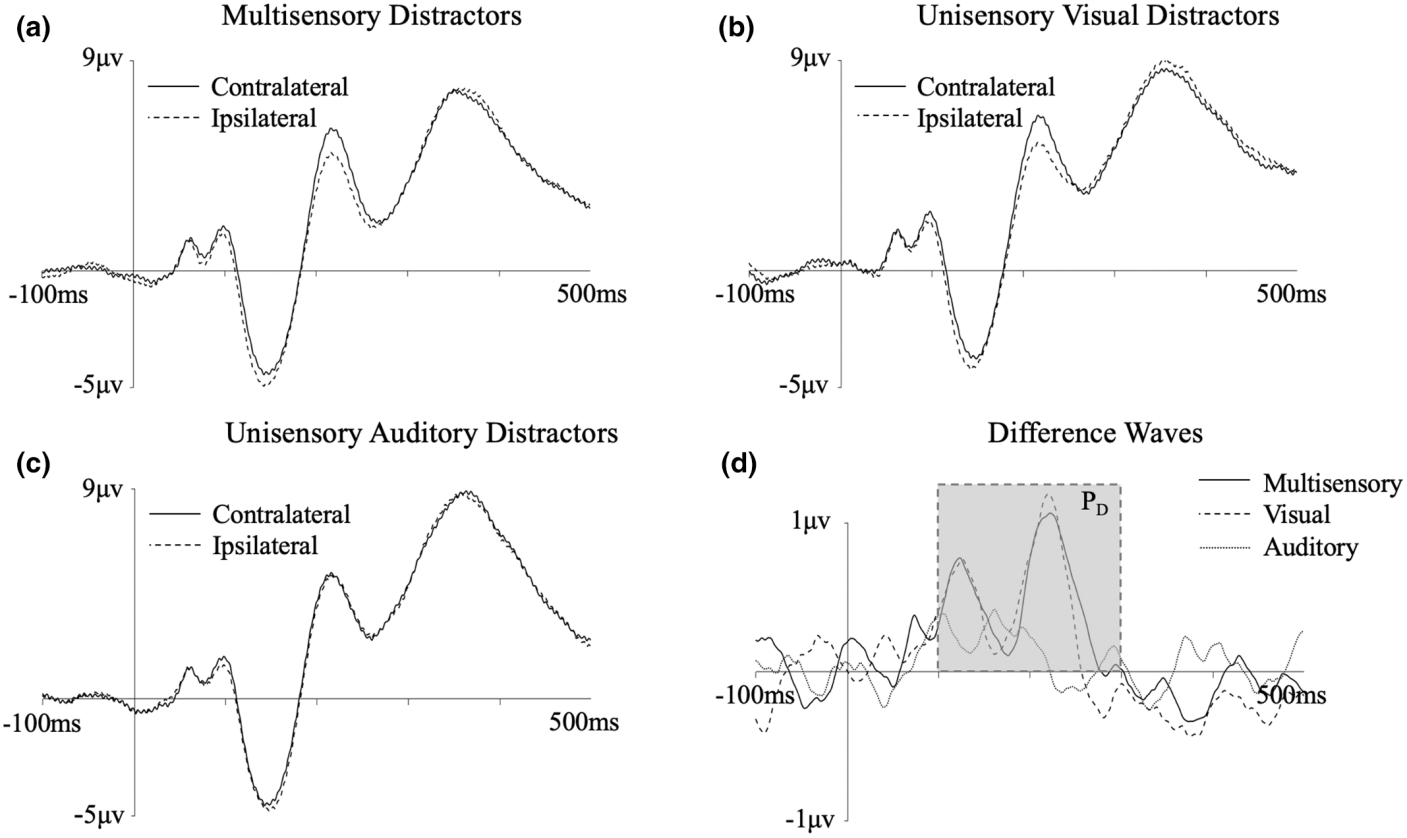
Grand averaged contralateral and ipsilateral ERP waveforms for (a) multisensory distractors (b) unisensory visual distractors, and (c) unisensory auditory distractors, and (d) contra‐minus‐ispilateral difference waves, at electrode sites PO7/PO8. The shadowed area represents the time window used for analysis of the P_D_.

**FIGURE 3:**
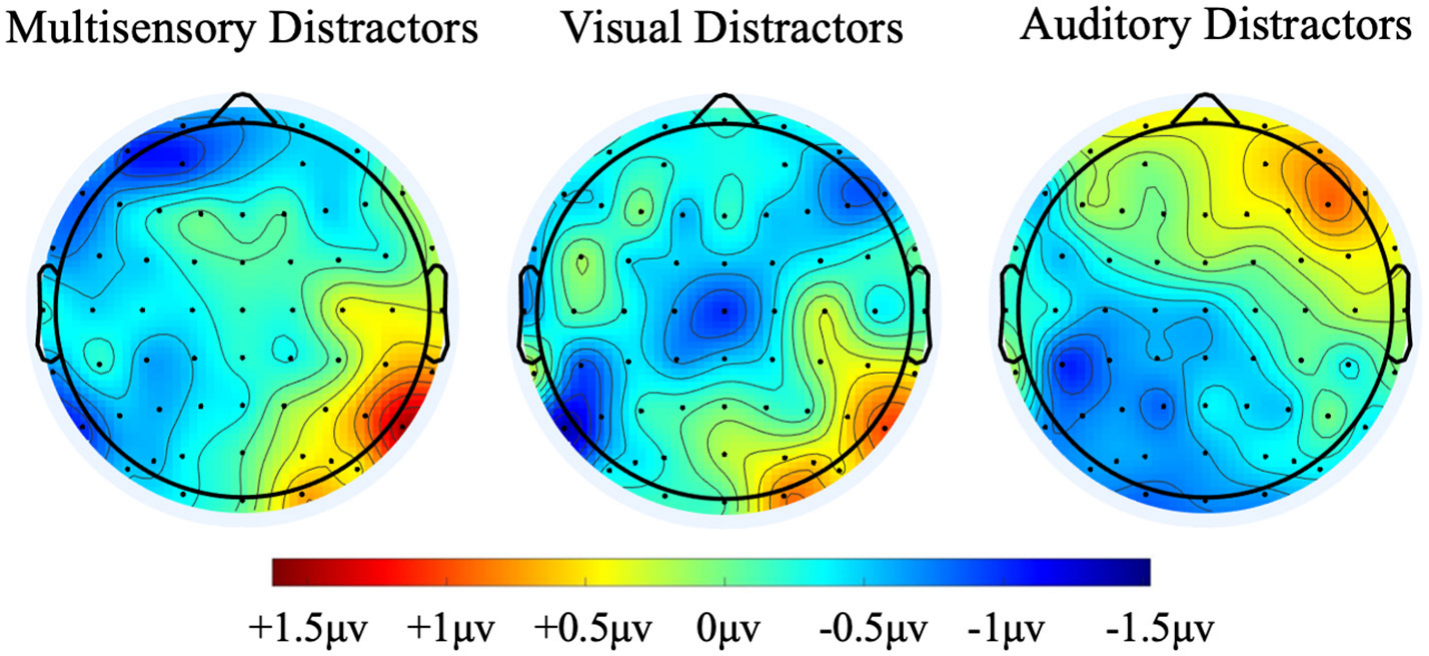
Topographic scalp maps of the P_D_ and P_AD_, based on mean voltage 100–300 ms, for the left‐minus‐right trial difference wave.

Figure 2 presents the grand averaged waveforms from lateral occipital scalp sites (PO7/8) for multisensory and unisensory distractors at contra‐ and ipsilateral electrodes, as well as the contralateral‐ipsilateral difference waves. As can be seen, there is no evidence for the presence of an N2pc, yet there is clear positivity at contralateral relative to ipsilateral electrode sites for the multisensory and unisensory visual distractors (see also Figure 3). An analysis of mean amplitude at 100–300 ms, chosen a priori, revealed a main effect of electrode laterality (*F*[1, 17] = 6.84, *p* = .018, η^2^ = .29), with contralateral waveforms more positive than ipsilateral waveforms, indicating the presence of a reliable P_D_ component. However, there was no significant interaction between laterality and distractor type (*F* < 1, η^2^ = .01), indicating that the P_D_ did not differ in size between multisensory and unisensory visual distractors.

**FIGURE 4:**
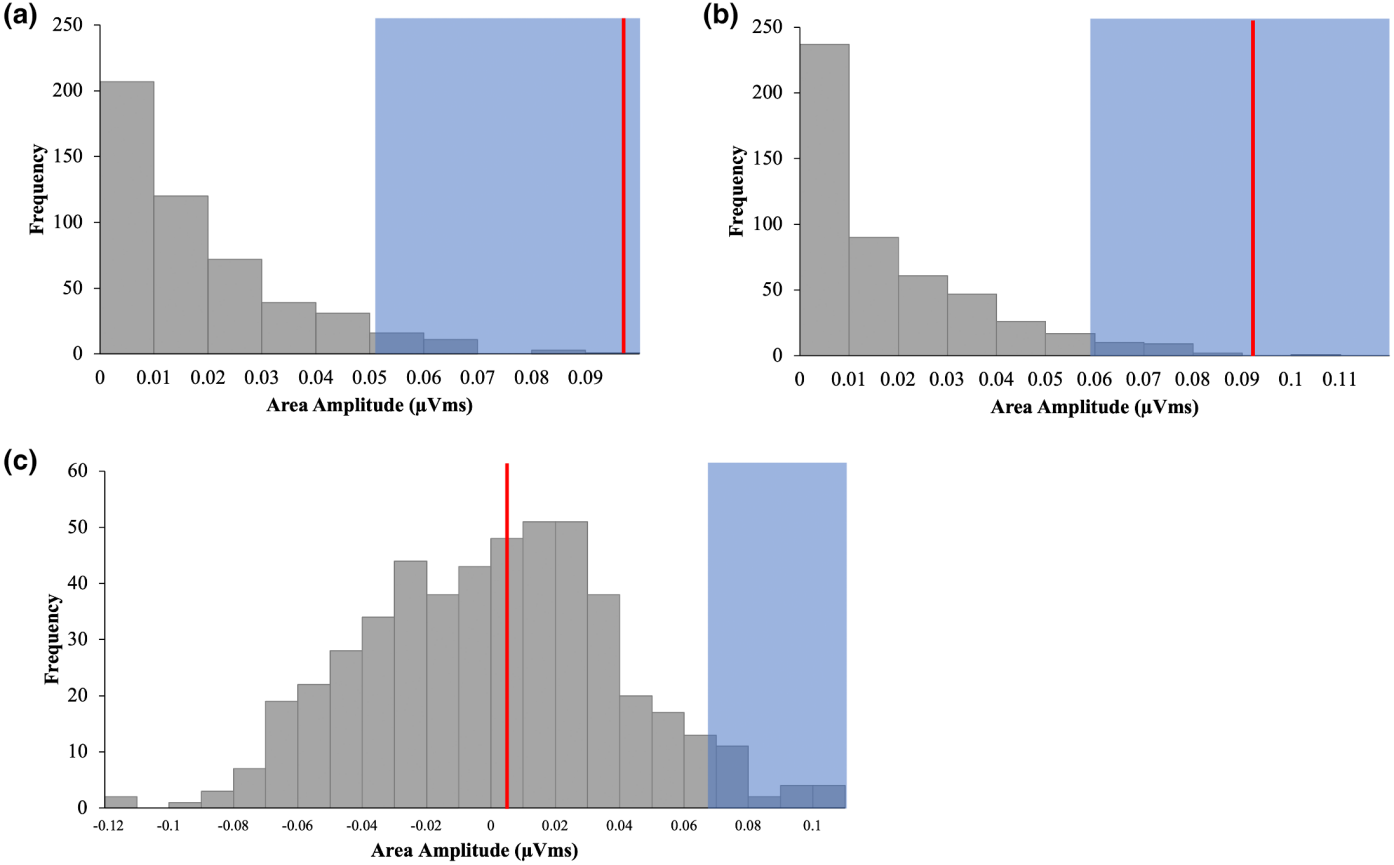
Observed value (red line) is significant if it falls within the top 5% of values of the permutation distribution (blue area). (a) Null distribution of multisensory distractors and observed significant value, (b) null distribution of visual distractors and observed significant value, (c) null distribution of interaction effect between distractor type (multisensory and visual), and laterality (contra, ipsi), and non‐significant observed value.

Our non‐parametric permutation analysis confirmed the above results. Figure 4 presents the null distributions of the multisensory and visual distractor conditions. For both distractor types the observed area (shown as a red vertical line) is greater than the 95th percentile of the null distribution (anywhere within the blue area would indicate this), and therefore can be taken as a significant P_D._ For the null distribution of the interaction effect (i.e., the difference wave of the multisensory and visual difference waves) the observed area (red line) is less than the 95th percentile of the distribution, and is confirmed to be non‐significant.

We note that visual inspection of the difference wave suggests that the P_D_ component for multisensory and visual distractors has two distinct peaks within our a priori time window: a peak of approximately 0.75 μV maximal at 125 ms, and a larger peak of over 1 μV which was maximal just under 100 ms later. Given discrepant findings as to whether the P_D_ may occur as early as 100 ms (e.g., Gaspelin & Luck, 2018), or whether this is more likely to be an early posterior contralateral positivity reflecting a sensory response (Ppc; Fortier‐Gauthier et al., 2012; Jannati et al., 2013), it was important to rule out any possibility of our P_D_ measurement being inflated by the Ppc. To this end, we repeated our analyses on a narrower time window which would capture the second peak only. A 175–250 ms time window was selected, consistent with that used by Gaspelin and Luck (2018), who similarly found this two‐peak waveform. An analysis of mean amplitude at this time in our data again revealed a main effect of electrode laterality (*F*[1, 17] = 11.48, *p* = .003, η^2^ = .40), and no significant interaction between laterality and distractor type (*F* < 1, η^2^ = .001). Non‐parametric permutation analysis using the same time window similarly found that the observed value of the P_D_ in both the multisensory and visual distractor conditions was greater than the 95th percentile of the null distribution (see Supplementary Materials for the full analysis). As such, an identical pattern of results was observed regardless of whether we use our a priori time window (which includes two peaks), or a time window that includes the second peak alone.

To test whether the active suppression occurs earlier for the multisensory distractors compared with the visual distractors, we examined the peak latency of both the early and late peaks, determined by the maximum positive amplitude in the contra‐minus‐ipsilateral difference wave at 100–150 ms and 150–300 ms. Neither peak occurred significantly earlier for the multisensory distractors (*M* = 124.83, *SD* = 15.23 for early distractors; *M* = 220.67, *SD* = 43.04 for late distractors) than the visual distractors (*M* = 127.56, *SD* = 16.39 for early distractors; *M* = 225.56 *SD* = 27.40 for late distractors; *t*s <1), indicating that the component occurred no earlier for the irrelevant multisensory stimuli.

#### 3.2.2 Auditory and multisensory distractors

**FIGURE 5:**
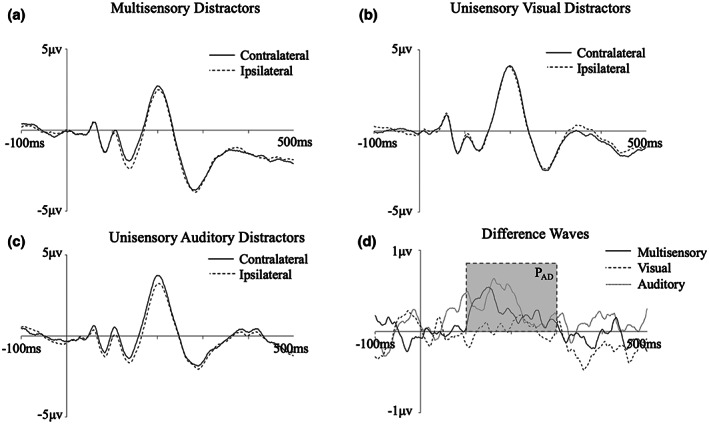
Grand‐averaged contralateral and ipsilateral ERP waveforms for (a) multisensory distractors, (b) unisensory visual distractors, and (c) unisensory auditory distractors, and (d) contra‐minus‐ispilateral difference waves, collapsed across electrode sites F3/4, F7/8, C3/4 and T7/8. The shadowed area represents the time window used for analysis of the P_AD_.

Figure 5 presents the grand averaged waveforms, across a cluster of anterior scalp sites (F3/4, F7/8, C3/4 and T7/8), for multisensory, unisensory visual, and unisensory auditory distractors at contra‐ and ipsilateral electrodes, as well as the difference waves. As can be seen, there is no evidence for the presence of an N2ac at this anterior electrode cluster, yet there is clear positivity at contralateral relative to ipsilateral electrode sites for the multisensory and auditory distractors, spanning approximately 100–300 ms (see also Figure 3). The location of this anterior positivity is inconsistent with the ACOP.

An initial mean amplitude analysis from 100–300 ms revealed a main effect of electrode laterality (*F*[1, 17] = 7.48, *p* = .014, η^2^ = .31), with contralateral waveforms more positive than ipsilateral waveforms. However, there was no significant interaction between laterality and distractor type (*F* < 1, η^2^ = .01). This suggests that there was an auditory equivalent of the P_D_ – indicative of spatial suppression of auditory distractors – for both conditions, and that this was of equal size between multisensory and unisensory auditory distractors.

**FIGURE 6:**
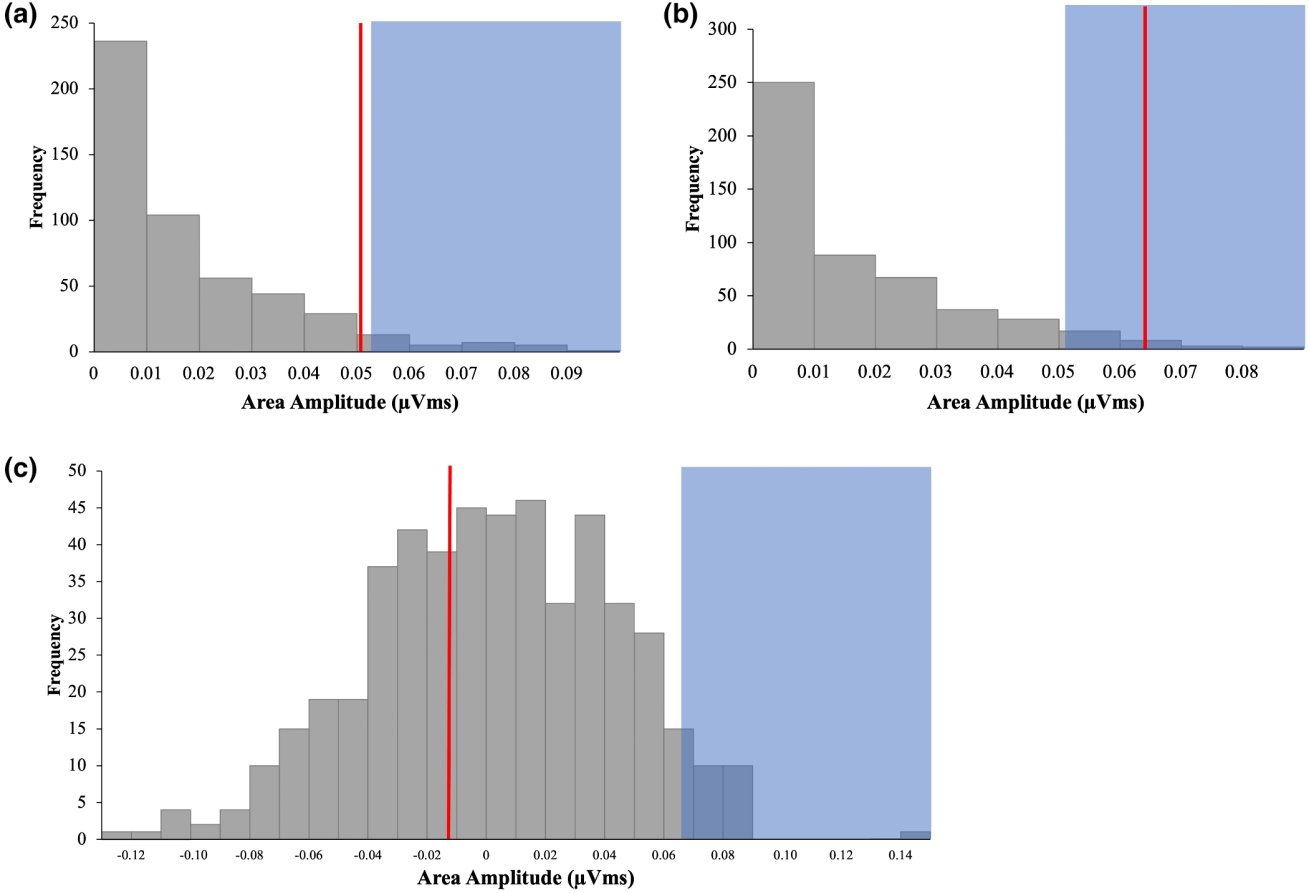
(a) Null distribution of multisensory distractors and observed marginally significant value, (b) null distribution of auditory distractors and observed significant value, (c) null distribution of interaction effect between distractor type (multisensory and auditory), and laterality (contra, ipsi), and non‐significant observed value.

The null distributions of the multisensory and auditory distractor conditions are shown in Figure 6. For the auditory distractor, the observed area (shown as a red vertical line) is greater than the 95th percentile of the null distribution (anywhere within the blue area would indicate this), and therefore can be taken as a significant P_AD._ The observed area for the multisensory distractor falls just short of the 95th percentile. For the null distribution of the interaction effect (i.e., the difference wave of the multisensory and auditory difference waves), the observed area (red line) is less than the 95th percentile of the distribution, and is confirmed to be non‐significant.

To test whether this component occurred earlier for the multisensory distractors than the auditory‐only distractors, we compared the peak latency of the contralateral negativity at the anterior electrode cluster, determined by the maximum positive amplitude in the contra‐minus‐ipsilateral difference waves between 100 and 300 ms. This showed that the auditory P_D_ did not occur significantly earlier for the multisensory distractors (*M* = 185.61, *SD* = 54.89) than the auditory distractors (*M* = 182.89, *SD* = 50.45; *t* < 1).

## 4 GENERAL DISCUSSION

The present study has three key findings. First, we demonstrate that entirely irrelevant visual distractors, appearing outside of the attentional window and sharing no features with the task stimuli, reliably elicited a P_D_ component. The P_D_ is argued to reflect suppression of a distractor stimulus in response to a spatial “attend‐to‐me” signal (Hickey et al., 2008; Sawaki & Luck, 2010). Second, we establish for the first time an auditory analog to the P_D_ component, henceforth referred to as the P_AD_: This novel component was observed in both of our task conditions involving auditory distractors (i.e., auditory unisensory and multisensory). Finally, we did not reveal any evidence for differences in spatial suppression of multisensory versus unisensory stimuli, in terms of either the time‐course or amplitude of the P_D_ /P_AD_ elicited by these stimulus categories.

As noted in our introduction, it has long been debated whether task‐relevance, through feature settings or location relevance, is necessary for attentional capture (e.g., see Folk et al., 1994; Theeuwes, 1992, 2010). The ability of our distractors to elicit the P_D_ and P_AD_ components imply that stimuli can compete for spatial attention, requiring active suppression to avoid distraction, even when they are entirely irrelevant to any top‐down goals associated with the task, and presented in an irrelevant location outside of the attentional window. In this respect our findings extend previous demonstrations that distractors in task‐irrelevant locations can elicit a P_D_ component (Neumann et al., 2018; Sawaki & Luck, 2010), by confirming this in a paradigm that can rule out both low level lateralized sensory effects and any relevance due to overlap in target‐distractor features (cf. Lichtenstein‐Vidne et al., 2012). Given the established sensitivity of the irrelevant distractor task to index the clinically inflated levels of distractibility seen in ADHD (Forster et al., 2014; Forster & Lavie, 2016), and taken together with evidence that the both N2pc and P_D_ amplitudes correlate with the severity of ADHD symptoms in children (Wang et al., 2016), the suppression mechanism underlying the P_D_ in the present paradigm appears a potential candidate for the disruption underlying ADHD‐related distractibility.

In the present experiment, multisensory and visual distractors elicited two P_D_ peaks. This two‐peak P_D_ has been found in previous research, with varying interpretation: On one hand, an early posterior contralateral positivity (Ppc), argued to reflect a sensory response, has been found at similar latencies as our early peak (e.g., Barras & Kerzel, 2016; Fortier‐Gauthier et al., 2012; Jannati et al., 2013). On the other hand, Gaspelin and Luck (2018) argued that the early positivity reflected distractor suppression (i.e., the P_D_) rather than a sensory signal, demonstrating it to be significantly larger when the lateralised stimulus was a distractor, versus a target. Future research could similarly test whether our early peak reflects a distractor suppression by using our lateralised stimulus as a target rather than a distractor. Importantly, however, in the present experiment, an analysis on the late peak only showed the same pattern of results as a collapsed analysis of the full P_D_ time window. Therefore, any debate over the identity of the earlier positive peak does not impact our conclusions regarding the ability of irrelevant distractors to elicit a P_D_.

Our distractors did not elicit an N2pc or N2ac component, which might imply a limitation in the ability of irrelevant distractors to fully capture spatial attention. We note that this finding must be taken in the context that, unlike prior studies using this task, we did not detect a behavioral distractor effect. As such, our ERP findings and behavioral findings concur in suggesting that, in this version of the task, the suppression mechanism reflected by the P_D_ component was successful in overcoming full spatial attentional capture. However, a similar paradigm using irrelevant visual distractors that did find evidence of behavioral interference also found the P_D_ rather than N2pc component (Neumann et al., 2018). Taken together with the prior study of Neumann et al., our results suggest that the suppression mechanism underlying the P_D_ is deployed in response to irrelevant distractors, irrespective of whether a behavioral effect is observed. Sawaki and Luck (2010)'s signal suppression hypothesis proposes that salient stimuli elicit a spatial “attend‐to‐me” signal, but that this can be actively suppressed, preventing attention being fully allocated to it. This hypothesis has garnered support from a variety of sources (see Gaspelin & Luck, 2019 for a review), including electrophysiological (e.g., Drisdelle & Eimer, 2021; Gaspelin & Luck, 2018), behavioral (Chang & Egeth, 2019; Gaspelin et al., 2015), oculomotor (Gaspelin et al., 2017; Weaver et al., 2017) and single cell recording (Cosman et al., 2018) research. Our results complement these various lines of research, and are compatible with a signal suppression hypothesis interpretation that entirely irrelevant distractors interfere with task performance through eliciting such a signal, but not necessarily eliciting full spatial attentional capture (Gaspelin et al., 2017; Sawaki & Luck, 2010; Weaver et al., 2017, see also Luck et al., 2021).

The lack of a behavioral distractor effect across all distractor types in the current study is surprising given that it is well documented in previous research employing the irrelevant distractor paradigm (e.g., Cunningham & Egeth, 2018; Forster et al., 2014; Forster & Lavie, 2008a, 2011, 2014; He & Chen, 2010; Lunn et al., 2019) and we cannot attribute this to lack of sensitivity, given the high power to detect a well‐replicated irrelevant distractor effect (99% based on both the effect size from the largest sample size, N = 100 Forster & Lavie, 2016 for the irrelevant visual distractor task, and from our most similar study by Lunn et al., 2019). We speculate that the behavioral distraction effect may have been undermined by changes necessitated in order to measure the ERP components of interest. Specifically, the inclusion of scrambled versions of each distractor was necessary to rule out an account of any lateralised effect in terms of early sensory differences, with balancing of displays to equate for low level factors being typical of research where either the N2pc or P_D_ components are measured (e.g., Berggren & Eimer, 2018; Gaspar & McDonald, 2014; Hickey et al., 2008; Sawaki & Luck, 2013); However, this change meant that the distractor lost its status as a unique abrupt onset, and is likely to have reduced its salience (see Chang et al., 2021, for a discussion of the determinants of salience in displays of various size). As discussed above, however, the common pattern of ERP findings between the present study and Neumann et al.'s (2018) prior study, suggests that the presence or absence of a behavioral effect does not substantially alter the presence of a P_D_ component in the irrelevant distractor paradigm, as well as implying that the findings of the prior study were not due to a display imbalance.

The second key contribution of the present article is to establish a new ERP component: the P_AD_, an auditory analog of the P_D_ component. This extends work by Gamble and Luck (2011), who demonstrated an auditory analog of the N2pc – the N2ac. Like the N2pc, the N2ac is characterized by a comparable negativity contralateral to the target, but occurs at a lower amplitude and across a more prolonged time window relative to the N2pc, at a cluster of anterior electrodes. Interestingly, our observation of the P_AD_, at the same electrode cluster in which N2ac has previously been observed, was also lower in amplitude and more sustained relative to the P_D_. Gamble and Luck (2011) originally proposed a potential explanation of the reduced amplitude and longer duration of the N2ac, relative to the N2pc, in terms of differences between auditory and visual processing (the auditory system having reduced contralaterality and auditory attention showing a differing time course). Our finding of a similar pattern of differences between the auditory versus visual analogs of another component linked to spatial attention (i.e., the P_AD_ versus P_D_) lends further support to this proposal, facilitating a direct comparison of auditory versus visual processing through the inclusion of both visual and auditory stimuli within the same paradigm. Our research is not only, to the best of our knowledge, the first evidence of a P_AD_, but also suggests that even an irrelevant stimulus presented in a different sensory modality to the target also elicits an “attend‐to‐me” signal that requires active suppression to avoid distraction. Future research may focus on the extent to which the P_AD_ reflects a similar (or the same) suppression mechanism as the P_D_. For example, is the size of the P_AD_ also associated with individual differences in attentional control (Gaspar et al., 2016; Sawaki et al., 2017), and is the P_AD_ only elicited where participants successfully orient their gaze to the target before or without fixating on the distractor (Weaver et al., 2017)?

Finally, our results speak to an ongoing debate regarding the proposed special status of multisensory stimuli for attentional capture (e.g., Van der Burg et al., 2008). If stimuli presented concurrently in two sensory modalities must integrate in order to enhance attention, then determining the conditions under which successful integration occurs is essential. Whilst there are a number of studies demonstrating that this integration requires endogenous attention, there are others suggesting that integration is automatic and pre‐attentive (see Soto‐Faraco et al., 2019; Spence & Frings, 2019; ten Oever et al., 2016 for reviews). Our design allowed us to compare the time course of spatial suppression for multisensory (audiovisual) versus unisensory (auditory or visual) distractors, as well as testing for potential interaction between these components. Whilst a multisensory distractor elicited both a P_D_ and a P_AD_, these did not occur at a different latency, or at an increased amplitude, to those elicited by the individual unisensory components. This suggests that the two sensory modalities are suppressed through two independent processes, with no multisensory integration which would require an enhanced, or non‐linear, active spatial suppression. We note that our design did not allow for a comparison between the multisensory and the sum of unisensory ERPs due to all trials containing both a visual and an auditory stimulus; however, previous research that has done this has demonstrated a lack of non‐linearity, indicative of multisensory integration, for an unattended multisensory stimulus (Talsma et al., 2007; see also Pápai & Soto‐Faraco, 2017 for a similar argument based on behavioral methods). This finding also supports our previous research where a to‐be‐ignored multisensory stimulus did not result in greater distraction than a unisensory stimulus (Lunn et al., 2019). Additionally, the ERP study conducted by Talsma et al. (2007) examined the effect of endogenous attention on the sensory components (e.g., P1 and N1), thus our study extends this previous research by looking at those that reflect spatial attention processes. As such, our results imply that entirely irrelevant multisensory stimuli do not hold any special attentional status.

In conclusion, we demonstrate that both visual and auditory distractors require active spatial suppression even when completely irrelevant to a current task, and that we avoid distraction by multisensory stimuli through independent suppression mechanisms acting on the two sensory components. Our findings speak to two theoretical debates on the ability of task‐irrelevant stimuli to compete for spatial attention, and the role of task‐relevance in the special attentional status of multisensory stimuli, and point to the P_D_ and P_AD_ as potential indices of the disruption underlying clinical symptoms of distractibility.

## AUTHOR CONTRIBUTIONS

**Jessica Lunn:** Conceptualization; formal analysis; investigation; methodology; writing – original draft; writing – review and editing. **Nick Berggren:** Methodology; validation; writing – review and editing. **Jamie Ward:** Writing – review and editing. **Sophie Forster:** Conceptualization; methodology; supervision; writing – original draft; writing – review and editing.

## CONFLICT OF INTEREST

The authors confirm that there are no known conflicts of interest associated with this publication and there has been no significant financial support for this work that could have influenced its outcome.

## Supporting information


Supinfo S1

## Data Availability

The data that support the findings of this study are openly available in the Open Science Framework at http://doi.org/10.17605/osf.io/yv6rg.
