# Supplementary material for: Irrelevant sights and sounds require spatial suppression: ERP evidence
Source: Psychophysiology. 2022 Sep 17;60(3):e14181. doi: 10.1111/psyp.14181 (PMC10078104; doi:10.1111/psyp.14181)
Supplement: Supplementary file 1 — Supinfo S1 [file PSYP-60-0-s001.docx]

**Supplementary Materials**

**Additional Descriptive Data**

|  | Distractor Condition | | | |
| --- | --- | --- | --- | --- |
|  | Multisensory | Auditory | Visual | No Distractor |
| RT(ms)  % Error | 563 (29)  6.42 | 564 (32)  6.06 | 575 (32)  7.50 | 557 (27)  9.39 |

**Table S1: Mean RTs (SE in parentheses) and error rates (%) as a function of load and distractor type, for the first four blocks only**

**Non-Parametric Permutation Analysis**

To perform the permutation test on the second peak only, methods were identical to those in the main experiment, except that the positive area value was measured between 175-250 ms. The null distributions of the multisensory and visual distractor conditions are shown in Supplementary Figure 1. For both distractor types the observed area (shown as a red vertical line) is greater than the 95^th^ percentile of the null distribution (anywhere within the blue area would indicate this), and therefore can be taken as a significant P_D._

_
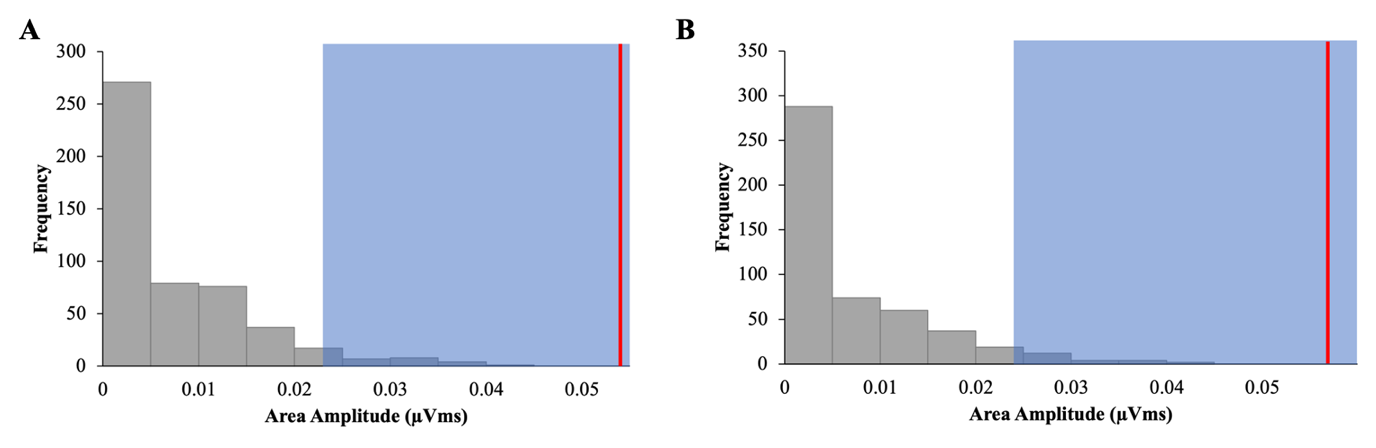
_

**SF1. A. Null distribution of multisensory distractors and observed significant value, B. Null distribution of visual distractors and observed significant value**
